# Resolving inconsistencies between Plötz’s descriptions and presumed type specimens of some Hesperiidae (Lepidoptera)

**DOI:** 10.3897/dez.70.98280

**Published:** 2023-04-03

**Authors:** Jing Zhang, Qian Cong, Leina Song, Jinhui Shen, Théo Léger, Gerardo Lamas, Olaf H. H. Mielke, Nick V. Grishin

**Affiliations:** 1 Department of Biophysics McDermott Center For Human Growth & Development, University of Texas Southwestern Medical Center, 5323 Harry Hines Blvd., Dallas, TX 75390, USA; 2 Department of Biochemistry McDermott Center For Human Growth & Development, University of Texas Southwestern Medical Center, 5323 Harry Hines Blvd., Dallas, TX 75390, USA; 3 Department of Eugene McDermott Center For Human Growth & Development, University of Texas Southwestern Medical Center, 5323 Harry Hines Blvd., Dallas, TX 75390, USA; 4 Museum für Naturkunde Berlin, Leibniz-Institut für Evolutions- und Biodiversitaetsforschung, Invalidenstr. 43, D-10115 Berlin, Germany; 5 Museo de Historia Natural, Universidad Nacional Mayor de San Marcos, Lima, Peru; 6 Departamento de Zoologia, Universidade Federal do Paraná, Caixa postal 19020, 81531-980 Curitiba, Paraná, Brazil

**Keywords:** biodiversity, classification, nomenclature, pseudotype, stability, taxonomy

## Abstract

Comparing specimens curated in MfN as primary types of Hesperiidae names proposed by Carl Plötz with the original descriptions and unpublished drawings reveals a number of inconsistencies that we address. Lectotypes are designated for *Telegonus labriaris* Butler, 1877, *Eudamus jalapus* Plötz, 1881, and *Apaustus interpunctata* Plötz, 1884. Neotypes are designated for *Netrocoryne seneca* Plötz, 1882 and *Hesperia irma* Plötz, 1882. *Hesperia ulphila* Plötz, 1883 is treated as a *nomen dubium*. As a result of these designations, the following are junior objective synonyms: *Netrocoryne seneca* Plötz, 1882 of *Telegonus labriaris* Butler, 1877 and *Hesperia irma* Plötz, 1882 of *Pamphila irma* Möschler, 1879, the latter two names being homonyms. In all these instances, the original descriptions are satisfied, and, except for *A. interpunctata*, the current application of these names is unaffected. Taxonomically, *Callimormus diaeses* Schaus, 1902, **stat. rest**. is a valid species, not a synonym of *A. interpunctata*, which is a junior subjective synonym of *Eutocus vetulus* (Mabille, 1883).

## Introduction

Primary type specimens serve as bearers of zoological names. They are essential to define each name objectively, connecting it to actual specimens that can be studied and compared to others using phenotypic characters or DNA sequences. With the recent advent of genomic sequencing successfully applied to century-old Hesperiidae type specimens (Cong et al. 2021), it becomes particularly important to ascertain that the specimens curated as primary types in collections are indeed the type specimens of the names they are considered to be types of.

Mistakes can occur in the original descriptions of taxa or during the curation and labelling of type specimens. Some mistakes may be revealed as inconsistencies between the original descriptions and the appearance of specimens curated as types, including their label data. These errors need to be addressed and corrected to agree with the major principles of zoological nomenclature recorded in the ICZN Code (ICZN [International Commission on Zoological Nomenclature] 1999). The stability of nomenclature that calls for the preservation of the current usage, particularly for the widely known taxa, is an important consideration. However, it needs to be balanced against historical accuracy that calls for applying a name to the taxon it was intended for by the original author.

Carl Plötz [1814–1886] named a large number of Hesperiidae species in the course of several years (Plötz 1879a–c, 1881a, b, 1882a–d, 1883a–d, 1884a–i, 1985, 1886). Many of Plötz’s descriptions were given as parts of identification keys and were not detailed enough to differentiate these species from others. However, Plötz also illustrated most of the Hesperiidae he included in his keys. Unfortunately, the original set of these illustrations could not be located. However, copies of some of Plötz’s drawings were assembled by Godman for the species he could not confidently recognise and attribute to specimens in his collection (Godman 1907). This compilation of the drawings, which are not particularly accurate (it remains unclear whether the originals were more accurate), is stored in the library of the Natural History Museum, London, UK (BMNH). At least a second copy of some of these drawings was made because similar drawings, cut into small cards, are pinned among the specimens of corresponding species in the collection of the National Museum of Natural History, Smithsonian Institution, Washington, DC, USA (USNM).

Due to sketchy descriptions and (where present) illustrations and frequently lost or unrecognisable type specimens, many of the names proposed by Plötz are still poorly understood, and their application to species is hypothetical, largely following a comprehensive revision by Evans that still remains the major source of Hesperiidae identifications (Evans 1937, 1943, 1949, 1951, 1952, 1953, 1955). During an attempt to learn more about Plötz’s names and the species they were intended for, we stumbled upon a number of inconsistencies in the data. In particular, these involve discrepancies between original descriptions, illustrations, and specimens, including their label data. Here, we discuss and resolve a number of such cases from the perspective of zoological nomenclature.

## Materials and methods

The specimens were inspected and photographed in the following collections: Natural History Museum, London, UK (**BMNH**), Zoological Institute and Museum Greifswald (**ZIMG**), Museum für Naturkunde, Berlin, Germany (**MfN**), and Zoologische Staatssammlung München, Germany (**ZSMC**). Historical documents, such as unpublished drawings, were inspected in BMNH and USNM. Photographs of specimens and illustrations were taken with a Nikon D800 camera through a 105 mm macro lens and processed using Photoshop. Genomic sequencing and analysis were carried out using our developed protocols as previously described: general methodology, specifics of working with historical specimens, and phylogenetic tree reconstruction are detailed in the following three publications, respectively (Li et al. 2019; Cong et al. 2021; Zhang et al. 2021).

## Results and discussion

We present the analysis of discrepancies we stumbled upon dealing with the names proposed by Carl Plötz. In each case, we suggest a specific solution chosen as a compromise considering all information available to us. However, first, we discuss general considerations regarding numbers on labels of type specimens cited in original descriptions.

### Type specimens and specimen numbers

A label with a number is commonly placed on a specimen to enable its cataloguing and referencing. Some of such numbers or labels with numbers may be unique to a single specimen; others denote a series where every specimen in the series has a label with the same number. An example of an unusual specimen-unique numbering system would be the one adopted in the past in MfN. Specimens were divided into series (most of the series had only one to four specimens), and each series was assigned a unique number, but apparently, only one label with this number was printed and pinned to a single specimen in the series, which we call a “header” specimen. Other specimens were placed in the collection, arranged in columns, after (below) the specimen with the number, typically bearing no labels on their pins at all. At times, subsequent curators were adding labels to these label-less specimens with the same number as the header specimen (probably to avoid information loss when the collection is rearranged), but these labels usually appeared different from the original printed number labels. Therefore, there may be a special significance to the header specimens in each series, the specimen that actually bears the label with the number. Specimens with such numbers were cited by Plötz in his descriptions as “Mus. Berol. n.” (see below). An example of a number being the same for a series of specimens is the numbering of type specimens in syntypic series in USNM of the names proposed by Schaus and Dyar. For many of these species, there is more than one specimen with the same number labelled as “type”.

### Not all specimens with the same number are equal

Although, unless there is evidence to the contrary–see Dolibaina et al. (2014) for an example involving *Cumbre cumbre* (Schaus, 1902)–all specimens with the same number labelled as “type” for taxa named by Schaus and Dyar in the USNM collection should be considered syntypes from the perspective of the ICZN Code, not all these specimens were equal in the eyes of Schaus and Dyar themselves. As evidenced by the handwritten labels on these specimens, both Schaus and, to a lesser extent, Dyar, differentiated between the specimen they considered the “type” and other specimens in the series they identified as conspecific with the “type”. Schaus placed handwritten identification labels on the specimens of his new taxa, putting the genus name as the first line, the species name as the second, and wrote “Schs” in the third. However, for the specimens he considered types, the third line stated “type Schs”. No “type” word was written on the identification labels of other specimens of the syntypic series by Schaus. Sometimes there are two specimens marked by Schaus as types: male and female. The concept at the time was that each sex should have its type. Dyar was less consistent, but many numbered specimens of his taxa did not bear his identification label with the word “type”, although some did (“Type Dyar” in the third line), and sometimes more than one for each sex. Although all these specimens (even those without handwritten identification labels) have the same “Type No.” labels, it is clear which specimens were preferred as types by Schaus (and to some extent by Dyar), and this fact should be taken into account for lectotype designations.

### A unique specimen number does not imply holotype

In his works, after the new species names he proposed, Plötz regularly wrote the phrase “Mus. Berol. n.” followed by a number, presumably printed on a label of the specimen Plötz referred to. Although labels with these numbers are unique to a single specimen (i.e., unless a mistake has been made, only one specimen in MfN has a characteristic label with this number), the numbers themselves are generally not unique to specimens with such labels and denote entire series of specimens, usually from one (in which case both the label and the number are unique) to four, as listed in the handwritten catalogue of the collection that needs to be studied in each case. Sometimes (see sections below), not all specimens in a series with the same number are from the same locality. Due to these reasons, we suggest that a reference to a single MfN number in Plötz’s description should not be taken as the holotype designation. The word “type” or its equivalent was not used, and Plötz made it clear that some of his descriptions were based on more than one specimen but provided only one number. For instance, the description of *Hesperia ulphila* Plötz, 1883 detailed both male and female, describing differences between them (Plötz 1883b) (see a dedicated section below). However, only a single number was given: “Mus. Berol. n. 5426”. One can only speculate why a single number was referenced and other specimen(s) was (were) not: there should have been at least two specimens involved, one considered by Plötz to be a male and the other presumed female. It is either because other specimen(s) did not bear a number, or were not in the Berlin collections, or their numbers were omitted by mistake. It is equally likely that all these specimens were from the same series under the same number. Regardless of the reason, it is possible, and even likely, that in many other cases where a single specimen number is referred to by Plötz, the description was based on more than one specimen, and the assumption of the holotype should be avoided, as suggested in the ICZN Code Recommendation 73F.

We note that in a number of cases, Plötz mentioned two collection numbers in descriptions, apparently when he considered that specimens with different numbers, and therefore from different localities, were conspecific. For instance, the original description of *Goniurus pilatus* lists two numbers: 5068 (3 specimens from “Bahia”, per catalogue of the collection) and 5069 (1 specimen from “Suriname”); and *Goniurus velinus* refers to 5102 (2 specimens from “Bahia”) and 5103 (2 specimens from “Caracas”) (Plötz 1881b). Plötz did not mention the number of specimens for each collection number, and only the collection numbers were listed. We found the number of specimens (2 specimens for each) in the collection catalogue.

### Neotype designation for *Netrocoryne seneca* Plötz, 1882

**Figure 1.**
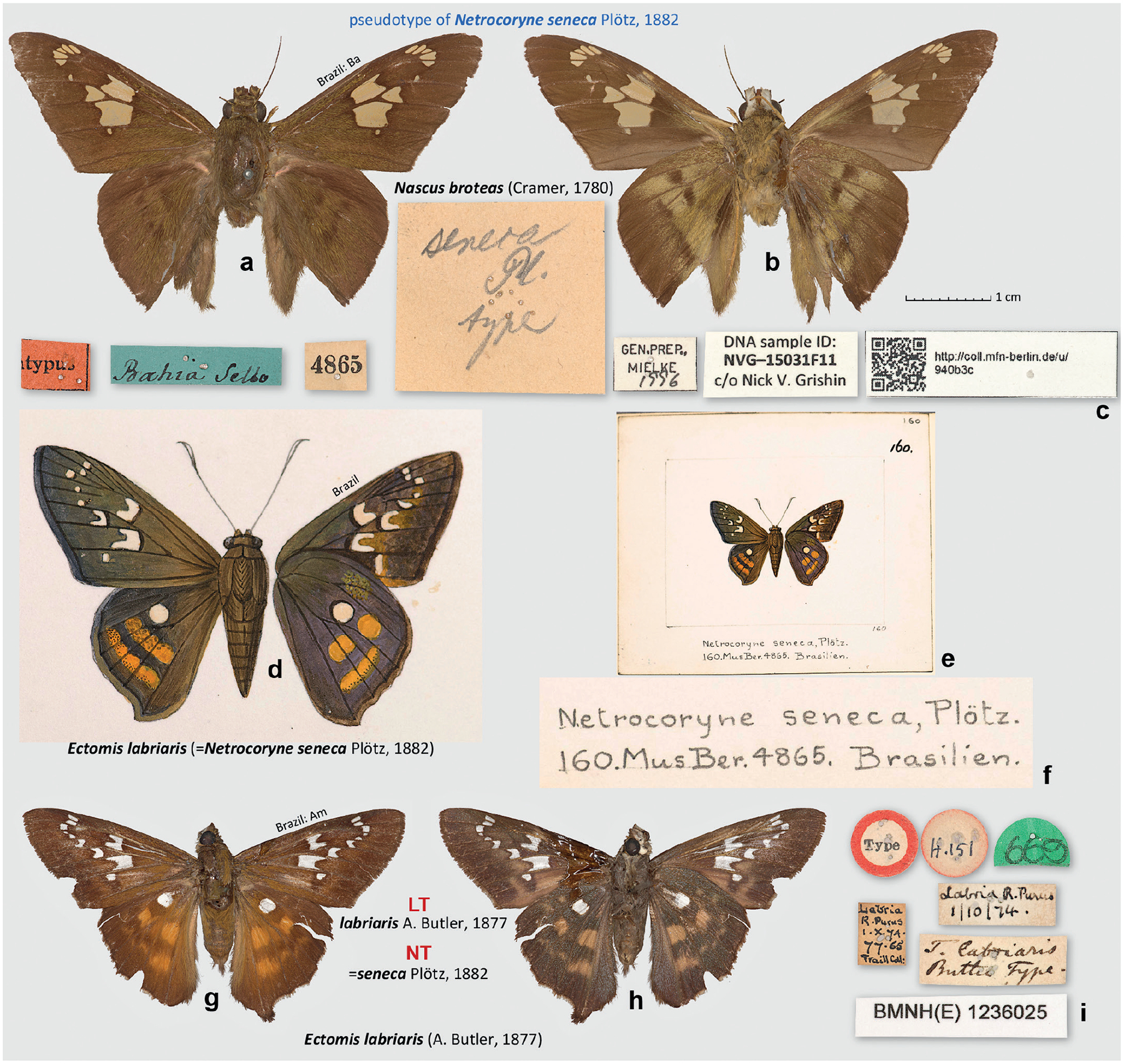
*Netrocoryne seneca* Plötz, 1882. **a–c**. Pseudotype of *N. seneca* with specimen number 4865 in MfN, which is *Nascus broteas*: **a**. Dorsal view; **b**. Ventral view; **c**. Labels; **d–f**. Godman’s copy of the unpublished Plötz’s drawing of *N. seneca* identifiable as *Ectomis labriaris*: **d**. Enlarged drawing to scale with specimen images (dorsal/ventral, left/right); **e**. Segment of a page with the drawing; **f**. Enlarged caption of the drawing; **g–i**. The lectotype of *Telegonus labriaris* Butler, 1877, which is simultaneously the neotype of *N. seneca*, both designated in this work: **g**. Dorsal view; **h**. Ventral view; **i**. Labels, the round label is shown from above (left) and below (right). All images are to scale, except **e**. Photographs **d–i**. (by N.V.G.) are Copyright of the Trustees of the Natural History Museum London and are made available under Creative Commons License 4.0 (https://creativecommons.org/licenses/by/4.0/).

In his description of *Netrocoryne seneca* (type locality Brazil), Plötz referred to “Mus. Berol. n. 4865” (Plötz 1882d). A specimen with this number (Fig. 1a–c) was found in the collections of the MfN, but it does not agree with the original description given by Plötz. The last segment of the key referring to *N. seneca* is translated as follows: “Forewing in almost all cells with small, disjointed hyaline spots; the across-dash-like [spot] in cell 1 is shifted towards the tornus, those [spots] in cells 2, 3 and the discal cell are deeply indented, there is no hyaline spot in the middle at the costal edge. The hindwing is only slightly elongated, with a round hyaline spot in the discal cell and 2 rusty yellow bands against the outer margin, cut by the dark veins; these bands are less complete on the underside. Colouring cloudy olive-brown, palpi grey. … t. 160. – Mus. Berol. n. 4865. [forewing length] 26 mm. Brazil.” This description is, however, a perfect fit for the unpublished drawing No. 160, copied by Godman’s decision, preserved in the library of BMNH (Fig. 1d–f).

Moreover, both the description and the drawing uniquely refer to a characteristically patterned and readily recognisable species known today as *Ectomis* (*Ectomis*) *labriaris* (A. Butler, 1877) (Li et al. 2019) (type locality Brazil: Amazonas, Lábrea, Rio Purus), which due to its unique appearance was once placed in a monotypic genus *Heronia* Mabille & Boullet, 1912 (Mielke 2005). The synonymy between *N. seneca* and *E. labriaris* had already been suggested by Draudt (1921–1924) and followed by all subsequent authors. The presumed “type” of *N. seneca* with the number 4865 on the label is *Nascus broteas* (Cramer, 1780) (type locality Suriname), which is patterned differently (e.g., it does not have even a trace of a hyaline spot in the hindwing discal cell), and therefore is not the specimen described and illustrated by Plötz. While we have not investigated the reasons for the inconsistency between the description reinforced by the illustration and the specimen with the number they are supposed to refer to, we conclude that the MfN specimen with the label “4865” is not a syntype of *N. seneca*.

To correct this problem, we searched for possible syntypes of *N. seneca* in the collections known to house Plötz’s types: MfN, ZSMC and ZIMG. The MfN collection catalogue lists two specimens for No. 4865, but we could not find this second specimen, which might have been the specimen Plötz based his illustration and description on. We also failed to find any other candidate specimens and believe that the syntypes of *N. seneca* are no longer extant. There is an exceptional need to stabilise nomenclature and define *N. seneca* objectively because the specimen with the number referred to in the original description is not *N. seneca*, creating a potential for nomenclatural instability if the identity of *N. seneca* is determined by this specimen. Moreover, the evidence we assembled is unambiguous in revealing the identity of the species that Plötz described as *N. seneca*, and we confirm the subjective synonymy between *N. seneca* and *E. labriaris* that has been in use for nearly a century. Therefore, we hereby designate a single known syntype of *E. labriaris*, female (Fig. 1g–i), as the lectotype of *Telegonus labriaris* Butler, 1877 and also as the neotype of *Netrocoryne seneca* Plötz, 1882, establishing that the latter is a junior objective synonym of the former.

This neotype of *N. seneca* satisfies all requirements set forth by the ICZN Article 75.3. Requirement 75.3.1: it is designated to clarify the taxonomic identity of this taxon, which may be threatened by a specimen of a different species curated as a syntype and bearing a label with the number referred to in the original description of *N. seneca*. Requirement 75.3.2: the characters for the taxon have been given in its original description by Plötz (1882d) and are re-stated above. Requirement 75.3.3: the neotype specimen bears the following labels: || Type || Labria | R. Purus | 1.X.74. | 77–65 | Traill Coll. || Labria R. Purus | 1/10/74. || T. labriaris | Butler Type. || 669 ||. Requirement 75.3.4: Our unsuccessful search for the syntypes of *N. seneca* is described above, leading us to conclude that they are lost. Requirement 75.3.5: as detailed above, the neotype is consistent with the original description and additional information (e.g., copies of Plötz drawings) known about this taxon and the current synonymy of this name. Requirement 75.3.6: the neotype is from Brazil: Amazonas, Lábrea, Rio Purus, and the type locality given for *N. seneca* in the original description is “Brasilien.” Requirement 75.3.7: the neotype is in the collection of the Natural History Museum, London, UK (BMNH).

### Lectotype designation for *Eudamus jalapus* Plötz, 1881

**Figure 2.**
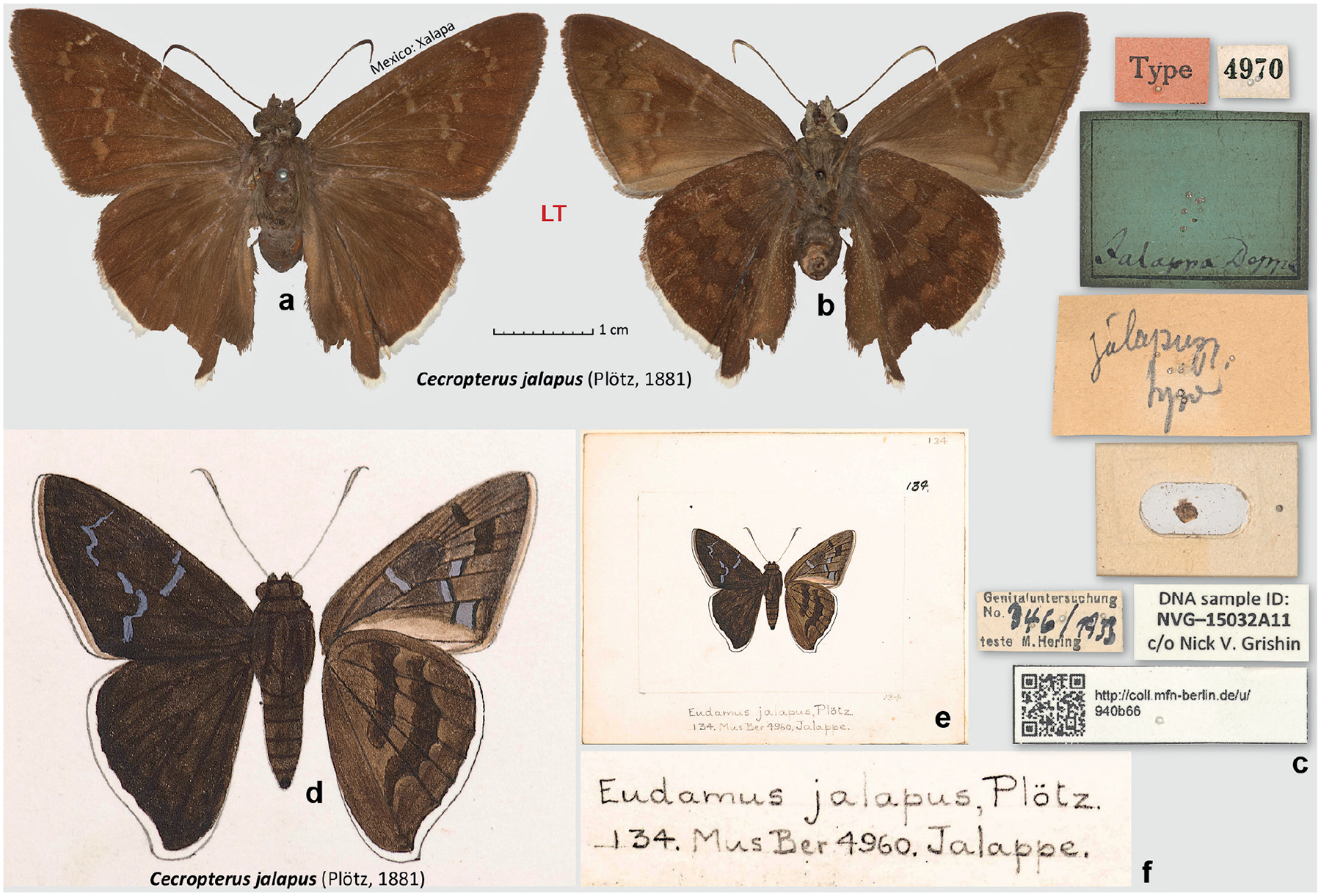
*Eudamus jalapus* Plötz, 1881. **a–c**. Lectotype of *E. jalapus*, currently in the genus *Cecropterus*, specimen number 4970 in MfN, designated in this work: **a**. Dorsal view; **b**. Ventral view; **c**. Labels; **d–f**. Godman’s copy of the unpublished Plötz’s drawing of *E. jalapus*: **d**. Enlarged drawing to scale with specimen images (dorsal/ventral, left/right); **e**. Segment of a page with the drawing; **f**. Enlarged caption of the drawing. All images are to scale, except **e**. Photographs **d–f**. (by N.V.G.) are Copyright of the Trustees of the Natural History Museum London and are made available under Creative Commons License 4.0 (https://creativecommons.org/licenses/by/4.0/).

In his description of *Eudamus jalapus* (type locality Mexico: Veracruz, Xalapa), Plötz referred to “Mus. Berol. n. 4960” (Plötz 1881a). However, the MfN catalogue lists a single specimen from Suriname identified as “*Hesperia creteus* var. *parmenides*”, and in the collection there is a *Telegonus* sp. from the *creteus* group with this number. Specimens with numbers close to 4960 are species of *Telegonus*. However, a specimen curated as a “Type” of *E. jalapus* had a number 4970 (Fig. 2a–c). For No. 4970, the MfN catalogue lists a single specimen of “*Hesperia* sp.” from “Jalappa” collected by Deppe. Turning to the original description of *E. jalapus*, we translate relevant parts of the key as: “Forewing without hyaline spots. Upperside dark brown. Forewing instead of the hyaline [spots] with several dark grey spots; underside of the forewing with dark spots, that [=underside] of the hindwing with 2 dark bands and a dark submarginal area … Fringes of the forewing whitish, those of the hindwing completely white. Underside violaceous-grey with more distinct [compared to *Cecropterus* (*Murgaria*) *albociliatus* (Mabille, 1877)] markings. Tornus of the hindwing at vein 1b pointed. … t. 134. – Mus. Berol. n. 4960. [forewing length] 26 mm. Jalappe”, which is an excellent match to the specimen 4970, except that its ventral side is more of a brown colour than “veilgrau” (i.e., “violaceous-grey”), and the forewing length is 27 mm.

The difference in colour was puzzling, so we turned to the unpublished drawing No. 134 by Plötz that was among those copied by Godman’s decision (Fig. 2d–f). The ventral hindwing colour on the drawing is more similar to the brown colour of the specimen No. 4970 than to our understanding of “veilgrau”. Speculations about the colour discrepancy include possible colour blindness, poor lighting in the room where descriptions were being made, broad application of the term “veilgrau”, or some mistake. To learn about Plötz’s use of veilgrau, we checked it in other instances. We found that veilgrau was indeed applied rather broadly to indicate shades from greyish (*Aguna asander* (Hewitson, 1867)) to brownish (*Polygonus savigny* (Latreille, [1824])), and it seems difficult to match an exact colour to veilgrau as used by Plötz. We conclude that the drawing No. 134 agrees well with the specimen No. 4970, and they both represent the name *E. jalapus* as it is applied currently, not calling for any taxonomic changes.

A more careful comparison of the specimen with the drawing suggests a possibility that this specimen No. 4970 might not be the specimen drawn as No. 134, because its size is larger, forewing fringes are darker, the forewing discal cell spot extends beyond this cell towards the costa, and the shape of the forewing spots differs somewhat. However, it is difficult to judge whether these differences are not merely inaccuracies of Plötz’s drawing or its Godman’s copy. The specimen No. 4970 fits the original description (corrected for the colour of the ventral hindwing) and specified type locality, carries a label with a number similar to the one indicated in the original description, and therefore we consider it is a syntype of *E. jalapus*. Moreover, this syntype, sequenced as NVG-15032A11, agrees with the current usage of this name. Therefore, to stabilise nomenclature, we designate the syntype in MfN, a female, bearing the following 7 labels: || Type || 4970 || jalapus | Pl. | type || Jalappa Deppe || Genitaluntersuchung | No. 346/1933 | teste M. Hering || \QR code\ http://coll.mfn-berlin.de/u/ | 940b66 || DNA sample ID: | NVG-15032A11 | c/o Nick V. Grishin || and a card with a genitalia capsule, as the lectotype of *Eudamus jalapus* Plötz, 1881, currently a species of *Cecropterus* Herrich-Schäffer, 1869 (Li et al. 2019). As for the discrepancy between the numbers indicated in the original description (4960) and pinned to the lectotype (4970), a likely explanation was a simple mistake when Plötz wrote 6 instead of 7.

### *Hesperia ulphila* Plötz, 1883 is a *nomen dubium*

**Figure 3.**
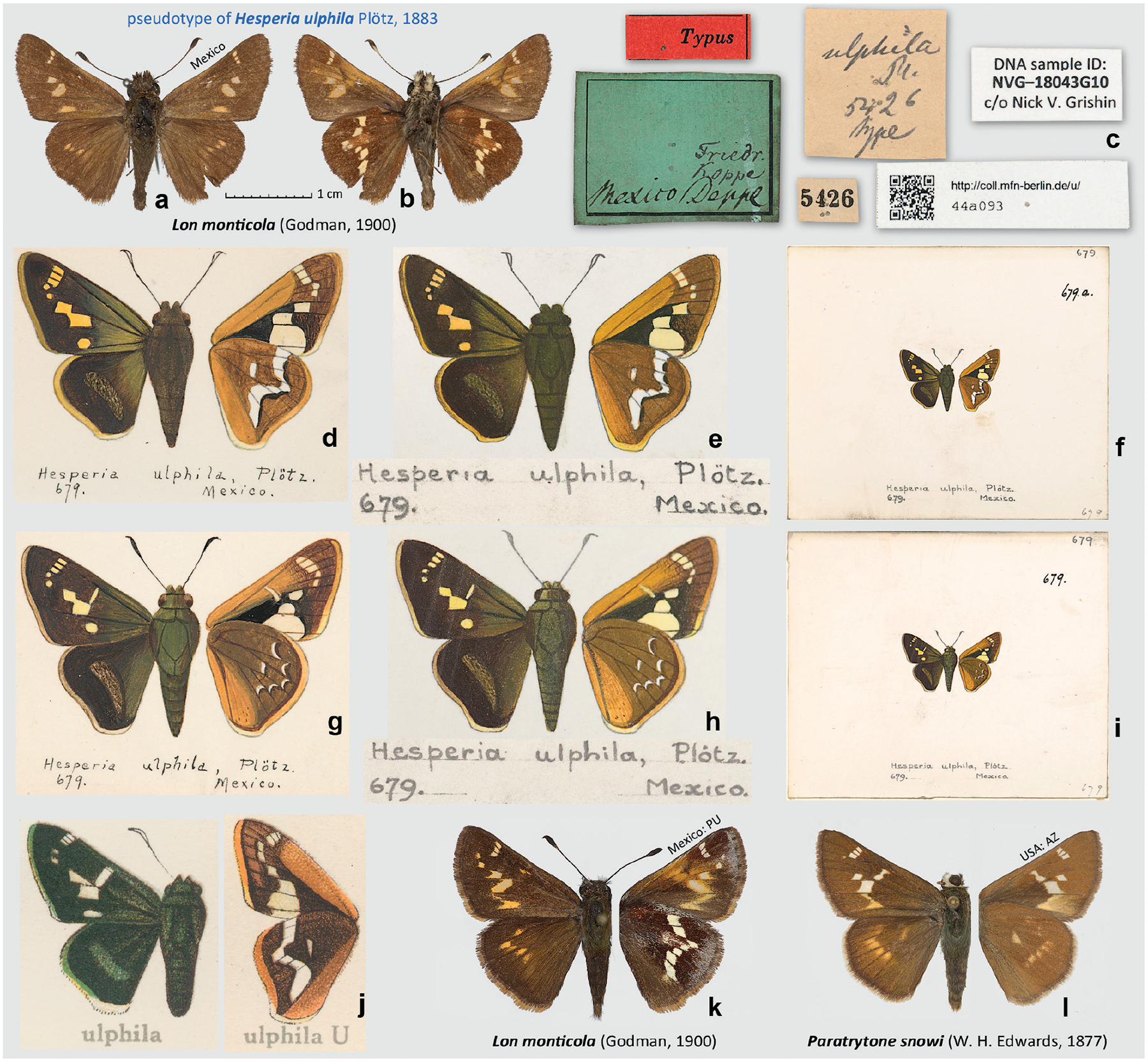
*Hesperia ulphila* Plötz, 1883. **a–c**. Pseudotype of *H. ulphila*, specimen number 5426 in MfN, which is *Lon monticola* (Godman, 1900): **a**. Dorsal view; **b**. Ventral view; **c**. Labels; **d–i**. Copies of the unpublished Plötz’s drawing of *H. ulphila*: **d–f**. Male; **g–i**. Female; **d, g**. Cards pinned in the USNM collection drawer with specimens; **e, f, h, i**. Godman’s copies in the BMNH booklet; **e, h**. Enlarged drawing to scale with specimen images, captions pasted below; **f, i**. Segments of pages with the drawings; **j**. Illustration of *H. ulphila* from the plate 182f [3, 4] in Draudt (1921–1924), a likely copy of Plötz’s drawing; **k**. *L. monticola* from Mexico: Puebla, Hwy 130, Puente Totolapa, ca. 8000 ft, 20°08’N, 98°06’W, 7-Jun-1992, J. Kemner [USNM]; **l**. *Paratrytone snowi* from USA: AZ, Coconino Co., San Francisco Mts., 6 km E of Humphreys Peak, 2440 m, 10-Aug-1991, M. Daman, R. Davidson, M. Klingler, W. Zanol, and J. Rawlins [CMNH]. Dorsal and ventral views are shown on the left and right, respectively. All images are to scale, except **f, i**. Photographs **e, f, h, i**. (by N.V.G.) are Copyright of the Trustees of the Natural History Museum London and are made available under Creative Commons License 4.0 (https://creativecommons.org/licenses/by/4.0/).

In his description of *Hesperia ulphila* (type locality Mexico), Plötz referred to “Mus. Berol. n. 5426” (Plötz 1883b). The specimen in the MfN collection with the No. 5426 (Fig. 3a–c) is curated as “Typus” of *H. ulphila*, but it shows discrepancies with the original description and Godman’s copies of Plötz’s drawings (Fig. 3d–f). Most significantly, the description states (as translated): “in cells 1–8 with the partly disconnected yellow spots … Underside … forewing … in cell 1 with large yellow [the drawing shows cream-coloured] spot. Hindwing … one cross-dash in the middle. … t. 679. – Mus. Berol. n. 5426. [forewing length] 16 mm. Mexico.” The “Typus” specimen is smaller (forewing length 14.5 mm, not 16 mm), it lacks well-developed spots in cells 4 and 5 (M_2_-M_3_ and M_1_-M_2_) as mentioned in the description and shown in the drawing, lacks a clear discal dash on the ventral hindwing, and the tornal area of the forewing is only somewhat paler than the ground colour, lacking a broad pale spot shown in the drawings (Fig. 3d–f).

Moreover, the drawing shows that the ventral hindwing discal band reaches the costal margin, and the spot in space 8 (C-Sc+R_1_) is present, making it three white spots anterad of the bend in the band (Fig. 3d, e, j), not two as in the “Typus” specimen (Fig. 3b), in which the band does not reach the costal margin and is separated from it by the brown-coloured cell C-Sc+R_1_. However, it is possible that the drawing is not particularly accurate because the hindwing cells 4 and 5 (M_2_-M_3_ and M_1_-M_2_) do not seem to be separated in the drawing, and only a single white spot is shown for them both (three spots caudad of the bend in the band, not four as expected). It is unclear whether the original drawing (not the Godman’s copies) was any more accurate, but the Draudt (1921–1924) illustration (Fig. 3j), which is unlikely to be drawn from the Godman’s copies, reveals a similar spot structure of the band: three spots anterad and three spots caudad of the bend, which is at a large spot, possibly illustrating both spots in cells 4 and 5 that do not appear to be separated from each other in the illustrated specimen. The discal band, the way it is drawn, has a similar spot structure in some specimens of *Metron zimra* (Hewitson, 1877) but is much narrower than in any *M. zimra* specimens we have seen.

In the case of *Eudamus jalapus* Plötz, 1881 (see above), the specimen curated as a type and bearing a label with a number different from that mentioned in the original description was consistent with the drawings and showed discrepancies with the description (ventral hindwing colour and specimen number). In the case of *H. ulphila*, the specimen curated as a type and bearing a label with the number mentioned in the description is inconsistent with both the original description and the drawing, which agree with each other. Therefore, we believe that the specimen No. 5426 in MfN, which is a species currently known as *Lon monticola* (Godman, 1900), is not a syntype of *H. ulphila*. As drawn, the presumed male *H. ulphila* may fit some of the better-patterned specimens of *L. monticola* that tend to develop pale spots in forewing cells 4 and 5 (Fig. 3k). Still, even such specimens lack a clearly defined dash in the hindwing discal cell (in them, it is more of a diffuse spot partly fused with the postdiscal band), the ventral hindwing discal white band does not reach the costal margin as in the drawing (although there is a possibility that the drawing may be inaccurate in this respect), and the tornal area of its ventral forewing is not broadly white as illustrated. Therefore, *H. ulphila* may be a different species waiting to be rediscovered.

Furthermore, we note that the MfN collection catalogue lists seven specimens in a series for the number 5426, all from Mexico, but collected by three different collectors: Friedrich, Koppe, and Deppe, thus not necessarily at a single locality, and could have included both males and females of several species. These specimens were not located in the MfN collections, there are no other specimens in the same column below the specimen bearing the label with No. 5426, and the green label mentions all three collectors (Fig. 3c). Therefore, these specimens are either lost or are among unlabeled specimens scattered throughout the collection. It is possible that only the header specimen was transferred from its original place to the drawer with type specimens, but the rest of the series, all six unlabeled specimens, might have remained in their original place. Checking many unlabeled specimens, N.V.G. was not able to find even one that closely agreed with illustrations of *H. ulphila*.

The presumed female specimen(s) of *H. ulphila*, mentioned in the description and illustrated by Plötz (Fig. 3g–i), has not been located either, and its taxonomic identity remains unclear. It differs from the male by having a dash in the forewing discal cell and the hindwing pattern consisting of 5 lunules in a semi-circle plus a discal cell dash. It may be a species different from the presumed male, and no species known to us fit the illustration well, particularly the forewing spots in cells 4 and 5 that were also causing identification problems for the male. However, with these two spots missing, a general appearance may be close to some specimens of *Paratrytone snowi* (W. H. Edwards, 1877) and relatives characterised by a broadly pale tornus of the ventral forewing.

Due to all these inconsistencies, and because species in two rather distantly related genera (*Lon* Grishin, 2019 and *Paratrytone* Godman, 1900) may at least partly agree with the copies of Plötz’s drawings, we propose to treat *Hesperia ulphila* Plötz, 1883 as a *nomen dubium*, and meanwhile search for specimens that fit the original description and drawings, more specifically, in having: (1) submarginal spots in forewing cells 4 (M_2_–M_3_) and 5 (M_1_–M_2_) and, ventrally, (2) a broadly pale forewing tornus and (3) a hindwing discal cell dash. These three characters seem to be shared by the presumed male and female *H. ulphila* syntypes as they were illustrated and conceptualised by Plötz (Fig. 3d–i). The main problem is that *Paratrytone* species tend to have a broadly pale forewing tornus, but they lack the spots in cells 4 and mostly in 5 (the specimen shown in Fig. 3l has a small dot on its dorsal side). The species of *Lon* may express the spots cells 4 and 5, but their forewing tornus beneath is not broadly white. We hope that our discussion and illustrations will facilitate the solution of this long-lasting puzzle about the identity of *H. ulphila*, a species that we are not even able to assign to a genus and that could potentially belong even to *Onespa* Steinhauser, 1974 or *Quasimellana* Burns, 1994, among others.

In summary, either the original drawings of *H. ulphila* (and, correspondingly, the original description, which was likely written from the drawings rather than actual specimens) contain a number of substantial inaccuracies, or *H. ulphila* is a lost species not currently among those with specimens known to us, and remains to be rediscovered, probably in southern Mexico or Central America.

### The specimen bearing a label with No. 5392 in MfN is not a syntype of *Apaustus interpunctata* Plötz, 1884

**Figure 4.**
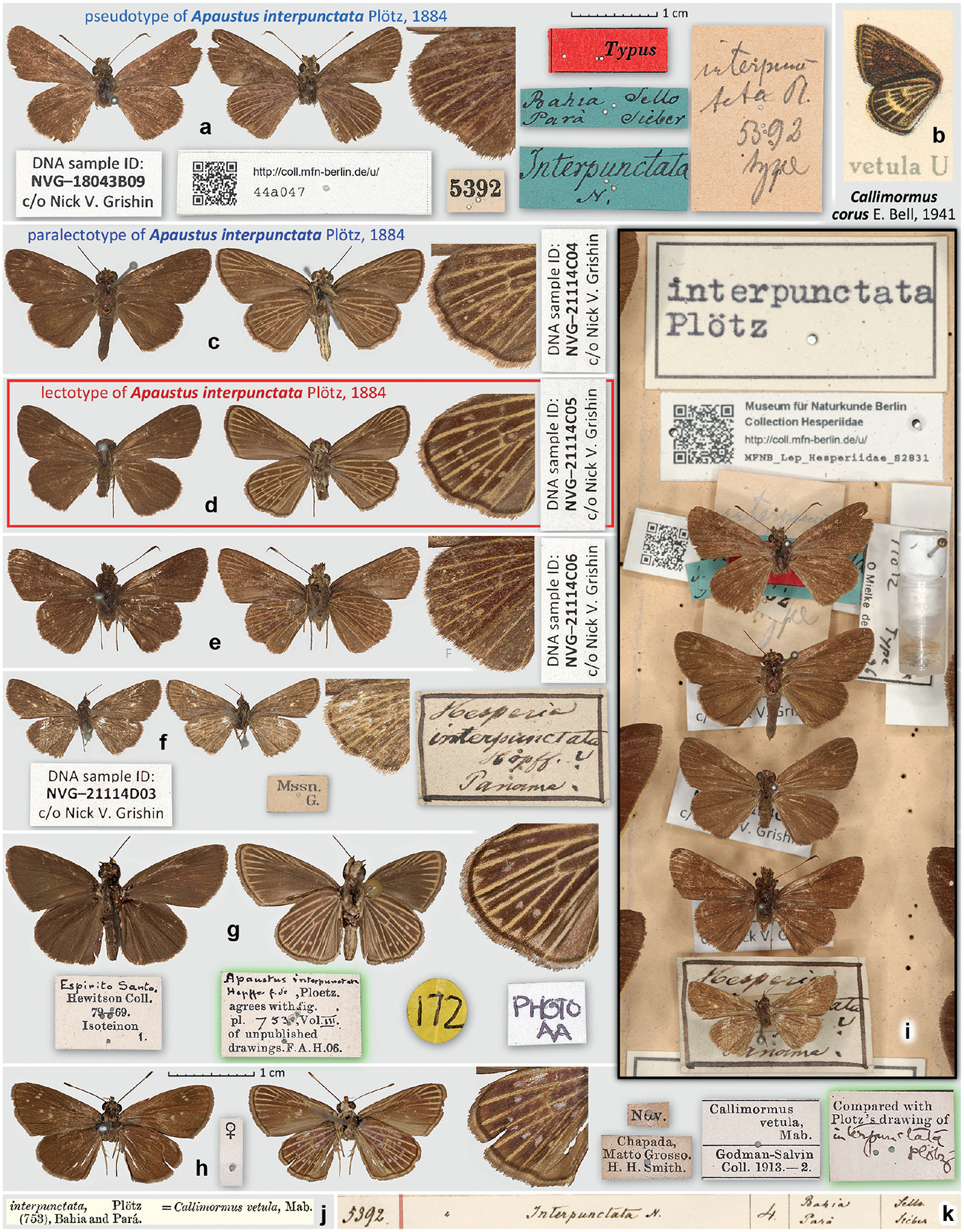
*Apaustus interpunctata* Plötz, 1884, specimens are separated by white lines, dorsal (left) and ventral (middle) views, enlarged hindwing venter (right), and labels (around) are shown for each specimen: **a**. Pseudotype, specimen number 5392 in MfN, *Vehilius vetula* (Mabille, 1878); **b**. Illustration of “*Callimormus vetula*” venter from the plate 189h [6] in Draudt (1921–1924); **c**. Paralectotype; **d**. Lectotype; **e**. Unlabeled specimen in the same column with the types; **f**. A specimen from Panama, originally from the collection of J. Peter Maassen; current identifications of these specimens are: **a, e**. *Vehilius vetula* (Mabille, 1878); **b**. *Callimormus corus* E. Bell, 1941; **c, d**. *Eutocus vetulus* (Mabille, 1883), and **f**. *Callimormus juventus* Scudder, 1872; **g, h**. Specimens in BMNH suggested looking similar to the original Plotz’s drawing No. 753 of *A. interpunctata*; **i**. A section of the Hesperiidae drawer in MfN with specimens identified as *A. interpunctata* (shown in panels **a, c–h**); **j**. Notes by Godman (1907) about the original drawing of *A. interpunctata*; **k**. An entry from the MfN collection catalogue for the number 5392. All images are to scale, except enlarged views of hindwing and texts. Gray F in **e**. indicates that the image is flipped (left-right inverted, left hindwing shown). Photographs **g, h**. (by Bernard Hermier) are Copyright of the Trustees of the Natural History Museum London and are made available under Creative Commons License 4.0 (https://creativecommons.org/licenses/by/4.0/).

In his description of *Apaustus interpunctata* (type locality Brazil: Bahia), Plötz referred to “Mus. Berol. n. 5392” (Plötz 1884c). The specimen in the MfN collection with the No. 5392 (Fig. 4a) is curated as “Typus” of *A. interpunctata*, but it shows discrepancies with the original description and would not be a good fit for the name itself, which apparently was derived from small pale dots (“inter”-”punctum”) between pale veins. The original description states (as translated): “Underside of the hindwing with 7 pale dots in a semicircle past the middle, veins pale, also [veins pale] towards the apex of the forewing. 27. *Interpunctata* Hpf. Mus. Berol. n. 5392. – Pl. t. 753. 12 mm. Bahia.” The specimen No. 5392 lacks these 7 pale “inter”-”punctum” (three weak spots are traceable at most) that formed the very basis of the name of this species. While descriptions may have mistakes of all sorts, and frequently the characters observed in a syntype would supersede those given in the description, in this case, the origin of the name strongly suggests that the description is accurate, but the specimen No. 5392 is not the one used to derive the name from and base the description on. Therefore, it is not a syntype of *A. interpunctata*, although it bears a label with the number 5392 given in the description.

### Investigations into *Apaustus interpunctata* Plötz, 1884, its lectotype designation, synonymy with *Eutocus vetulus* (Mabille, 1883) and reinstatement of *Callimormus diaeses* Schaus, 1902 as a valid species

Inspecting unpublished Plötz’s drawings, Godman (1907) (Fig. 4j) identified *A. interpunctata* as:

“*Callimormus vetula*, Mab.” by which he certainly meant the species known today as *Eutocus vetulus* (Mabille, 1883) (originally proposed in *Cobalus* Hübner, [1819]), not *Pamphila vetula* Mabille, 1878 (currently in *Vehilius* Godman, 1900). This is evidenced by the identification labels on specimens in Godman and Salvin collection, now in BMNH, one of them shown in Fig. 4h. Because Godman found a specimen in his collection that was sufficiently similar to the original Plötz’s drawings of *A. interpunctata* (no. 753), he did not select this drawing to be copied. Instead, he placed a label on the specimen that was printed with “Compared with Plötz’s drawings of”, followed by a handwritten Plötz’s species epithet, a practice that he followed in such cases (Fig. 4h label highlighted in green). Therefore, this specimen, shown in Fig. 4h, could be used for assessing how the original Plötz’s drawing looked like. This specimen also agrees with the original description of *A. interpunctata* without any discrepancies. This specimen from Brazil: Mato Grosso is a paratype of *Eutocus matildae vinda* Evans, 1955 (type locality in Peru: Amazonas), currently a junior subjective synonym of *E. vetulus* as determined by Mielke and Casagrande (2002).

Apparently, Godman was not the only one who studied the original Plötz’s drawings at the time when they were in London. In 1906, just a year before Godman’s paper, Francis Arthur Heron (1864–1940), who was an assistant curator in the BMNH, found a different specimen that “agrees with fig. [sic!] pl. 753, Vol. III. of unpublished drawings.” (Fig. 4g label highlighted in green). This label also reveals that Plötz’s drawings were bound in at least three volumes. The specimen selected by Heron is similar in general appearance to the one chosen by Godman and additionally reinforces the idea about how *A. interpunctata* looked like. However, it is a less precise match to the original description because only 6 dots (unless a streak in cell CuA2-1A+2A is counted as a dot) are present on the ventral hindwing: the dot in the cell M_2_–M_3_ is not expressed. This specimen was later identified by Evans as *Eutocus fabulinus* (Plötz, 1884). Thus, due to superficial similarities, two different workers selected specimens of two different species as possible candidates for how *A. interpunctata* might have looked.

Draudt (1921–1924) reproduced Plötz’s drawing, frequently losing in quality, as illustrations of species that were not figured in other sources, and he was not able to get specimens of (e.g., *Hesperia ulphila* Plötz, 1883, Fig. 3j). Likely following Godman, Draudt listed synonymy with *A. interpunctata* and illustrated his concept of “*Callimormus vetula*” (i.e., *Cobalus vetulus* Mabille, 1883, not *Pamphila vetula* Mabille, 1878, which he treated as “*Phlebodes vetula*”) (Fig. 4b). This illustration depicts neither *Eutocus vetulus* nor resembles specimens in BMNH selected to represent *A. interpunctata* (Fig. 4h, g), but is identifiable as *Callimormus corus* E. Bell, 1941 (type locality Brazil: Pará) due to the pale veins by the forewing costa and the hindwing spots nearly fused into a band. Therefore, Draudt’s illustration is not likely to be a copy of Plötz’s drawing of *A. interpunctata* and may not offer additional clues about its identity.

Then, we turned to the catalogue of historical specimens in MfN. This catalogue consists of several handwritten notebooks with handwriting matching that of Carl Heinrich Hopffer (1810–1876), who was the curator of the entomological collections in the MfN until his death. The catalogue lists species and their localities by their collection numbers, one number per line. If there is more than a single locality for one specimen number, the next locality is listed under the previous. The entry for No. 5392 states: 5392 [collection number] \ Interpunctata N. [species name followed by an unknown abbreviation] \ 4 [number of specimens] \ Bahia / Pará [locality] \ Sello / Sieber [collectors] (Fig. 4k). It means that four specimens are listed under the number 5392, but only one of these specimens, the “header” specimen, is expected to bear the original printed label “5392” on its pin. The meaning of “N.” following “Interpunctata” is not totally clear. Specimen identification labels of similar style and handwriting (i.e., of Hopffer) are found throughout the collection (e.g., Fig. 4a). For the names already published, Hopffer gives the author’s name abbreviation and cites the reference. The names published after his death (1876) by others, e.g., Plötz, or unpublished, are followed by this “N.” It is not likely to be an abbreviation of a person’s name, but it could stand for the Latin Nobis, i.e., “us” or “of us”, the same as “m.” would mean “mihi”. i.e., “me” or “of me.” Alternatively, and possibly more likely, “N.” could be an abbreviation for the Latin “Nova” or German “Neue”, simply meaning that the name or species was new. The origin of these names with “N.” is also unclear. The names could have been provided to Hopffer by Plötz, who had already prepared the drafts of his manuscripts with names and drawings before 1876, or the other way around, coined by Hopffer himself and simply used by Plötz after Hopffer’s death.

The segment of the drawer with the “Typus” specimen with the 5392 label, which is a pseudotype of *A. interpunctata*, is shown in Fig. 4i. There are three similar-sized brown specimens in the same column under it. These specimens did not have any labels at the time they were located in the drawer. It is most likely that these 4 specimens under the header label [interpunctata | Plötz] are the four specimens listed in the collection catalogue. Therefore at least some of them were available to Plötz for his study. The header specimen (the pseudotype) bears a locality/collector label with the same wording and in the same handwriting as in the catalogue: [Bahia Sello | Pará Sieber] and the same name label [Interpunctata | N.] (Fig. 4a green labels). We hypothesise that these three labels: the number, locality, and name, were the original labels, and they refer to all 4 specimens and not just to the pseudotype header specimen. This explains the two localities (and collectors – Friedrich Sello[w] [1789–1831] and Friedrich Wilhelm Sieber [1775–1831]) on the label. It is likely that some of these 4 specimens were from one locality, and some were from the other. Other labels were added at some later time, and the label [interpunct- | tata Pl. | 5392 | type] is in a different handwriting than the original labels, and because it mentioned the word “type” may not have been written by a German (Fig. 4a).

There is the fifth specimen within this *interpunctata* block (Fig. 4i). It is smaller than the four, labelled from Panama, comes from J. Peter Maassen collection (hence collected before his death in 1890) and is identified as “Hesperia interpunctata Hopff[er]. ?” It is not a possible syntype of *A. interpunctata* because it is from Panama, not from Bahia, but we discuss this specimen here because Plötz also attributed the name *interpunctata* to Hopffer (as Hpf.). However, the identification label might have been written using Plötz’s publication, not before it, when Maassen was attempting to identify his specimens, and the question mark indicates that he was unsure of this identification. In any case, it is likely that this specimen was added to the four original specimens at a later date, maybe when the Maassen collection was being incorporated into MfN holdings and was not mentioned in the original collection catalogue.

**Figure 5.**
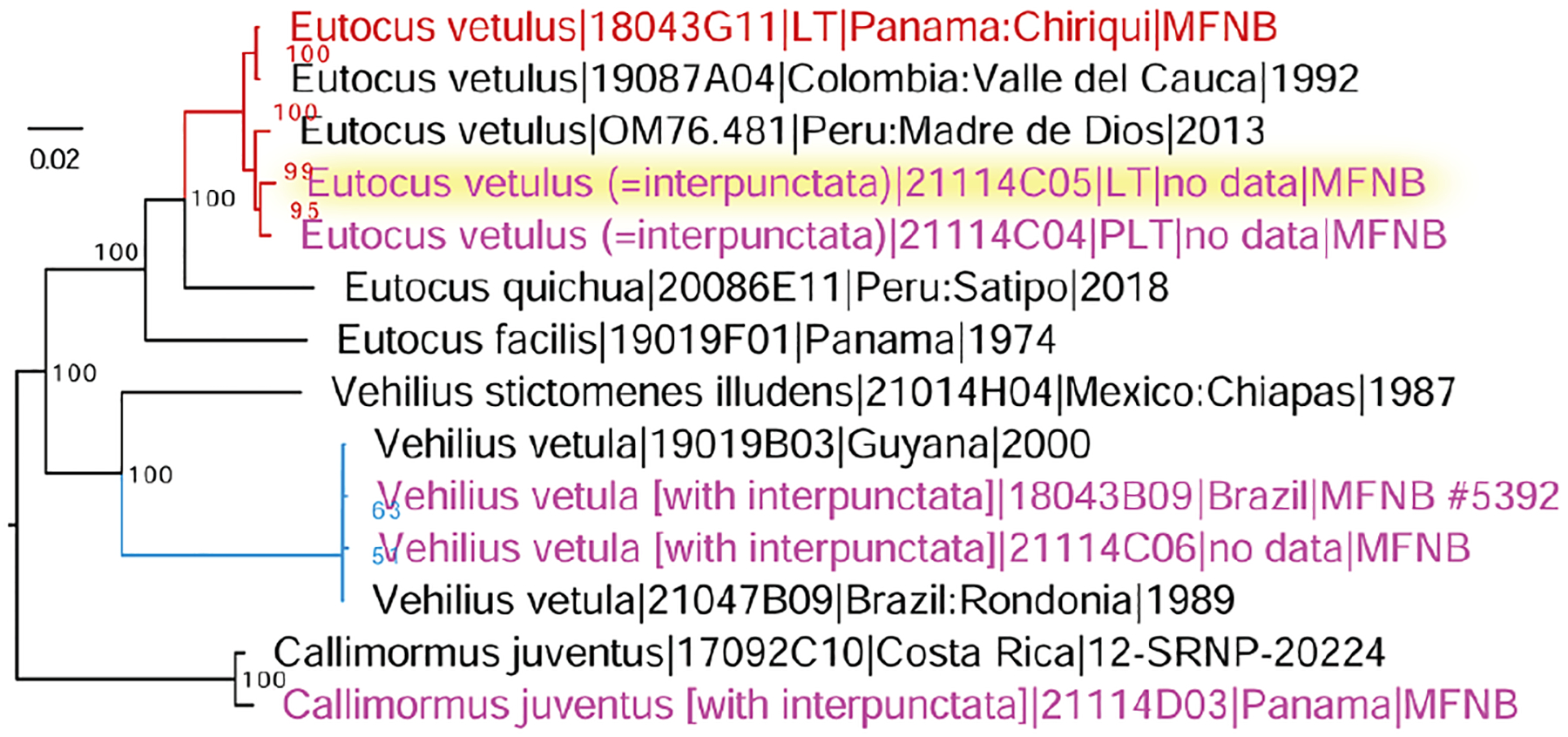
Mitochondrial genome tree of *Eutocus vetulus* (red), *Vehilius vetula* (blue), and relatives. Five specimens in MfN identified as *Apaustus interpunctata* are labeled in magenta and the lectotype is highlighted in yellow. The lectotype of *E. vetulus* is labeled in red.

Legs of all five specimens in MfN identified as *A. interpunctata* were sampled for genomic sequencing, and sample ID labels were added to them (Fig. 3a, c–f). Thus, the three originally unlabeled specimens gained the DNA sample labels (Fig. 3i). The genomic datasets of these specimens were compared with others using phylogenetic trees, and a tree constructed from all protein-coding regions of the mitochondrial genome (good to illustrate partitioning into species) is shown in Fig. 5. Both by genomic analysis and by phenotype, and using current taxonomy, 1^st^ (Figs 4a, 5 NVG-18043B09) and 4^th^ (Figs 4e, 5 NVG-21114C06) specimens are *Vehilius vetula* (Mabille, 1878), 2^nd^ (Figs 4c, 5 NVG-21114C04) and 3^rd^ (Figs 4d, 5 NVG-21114C05) specimens are *Eutocus vetulus* (Mabille, 1883), and the 5^th^ specimen (Figs 4f, 5 NVG-21114D03, from Panama) is *Callimormus juventus* Scudder, 1872 (type locality in Panama). Identification of *E. vetulus* is supported by sequencing of its lectotype designed by Mielke and Casagrande (2002), from Panama, also in MfN (Fig. 5 NVG-18043G11). We see that the four original specimens, including the pseudotype, belong to two distinct species in two different genera. These species share a homonymous species epithet, which, curiously, was proposed by the same author: Paul Mabille, and caused some confusion, as detailed by Mielke and Casagrande (2002).

The locality label for the four specimens belonging to two species gives two distinct states in Brazil: Bahia and Pará (Fig. 4a). It is tempting to speculate that two specimens of one species were collected at one locality and two specimens of the other species were collected at the second locality. Because the *V. vetula* type locality is in Pará, it is probable that more specimens of this species were collected around the same time at the same locality. Therefore, it seems likely that the two *V. vetula* specimens (not agreeing with the description of *A. interpunctata*) were collected in Pará. Hence, the two other specimens, which are *E. vetulus* (agreeing with the description of *A. interpunctata*), were probably collected in Bahia. Notably, only Bahia is mentioned as the locality in the original description, supporting our line of thought. Thus, we suggest that the two *E. vetulus* were collected in Bahia by Sello and not in Pará by Sieber.

We note that the original Plötz’s drawing listed both localities (“Bahia and Pará”) per Godman (1907) (not clear if more than one specimen was illustrated or just one with two localities given for it), thus Plötz was aware of both localities at the time he was making the drawings, but chose only one locality in his description. We can only speculate that initially, Plötz might have considered all these four specimens to be conspecific, and those lacking all 7 dots (i.e., *V. vetula* from Pará) were assumed to be worn or variatons. However, when Plötz wrote the description, he probably restricted the concept of *A. interpunctata* to the specimens with 7 dots (i.e., *E. vetulus*) and thus to Bahia only. These speculations suggest that the two *V. vetula* specimens were not syntypes (i.e., Plötz did not consider them to be *A. interpunctata* at the time of his publication). A problem arose when the four specimens were still curated under the same original number in the MfN collection (because nobody re-curated them after Plötz’s description). The header specimen was still bearing the label with the number listed in the original description of *A. interpunctata*, but Plötz probably no longer considered this header specimen of the series that bears that number to be conspecific with his *A. interpunctata*. After the description was published, curators placed the red “Typus” label, either directly on this header specimen with the number mentioned in the description or as another header label to the series (as the green Bahia/Pará label) that later found its way on the pin of the header specimen when the collection was under curation.

We realise that our line of thought above is highly speculative, but the facts are: (1) two *E. vetulus* specimens out of four specimens in “Mus. Berol.” series “n. 5392” (per collection catalogue) fully agree with what is known, published and unpublished, about *A. interpunctata*. (2) two *V. vetula* specimens out of four specimens in “Mus. Berol.” series “n. 5392”, including the header specimen, do not agree with the original description and specimens in BMNH selected as the best match to the original drawing of *A. interpunctata*. For these reasons, the two *E. vetulus* specimens are syntypes of *A. interpunctata*, and we hereby designate the specimen with better developed 7 hindwing dots and thus in better agreement with the original description, illustrated in Fig. 4d and currently bearing a single label [DNA sample ID: | NVG-21114C05 | c/o Nick V. Grishin] as the lectotype of *Apaustus interpunctata* Plötz, 1884 (type locality Brazil: Bahia) to stabilise nomenclature. This specimen lacks antennae and, likely due to dermestid damage, the distal part of its abdomen. The second specimen (NVG-21114C04) is a paralectotype.

In summary, we support the original synonymy suggested by Godman (1907) and, by the identity of its lectotype, regard *Apaustus interpunctata* Plötz, 1884, syn. rev. as a junior subjective synonym of *Eutocus vetulus* (Mabille, 1883) (has ventral hindwing discal cell pale spot(s)). As a result, *Callimormus diaeses* Schaus, 1902, stat. rest. (lacks ventral hindwing discal cell pale spots), which is the only available name for the species that Evans (1955) misidentified as *Callimormus interpunctata* (Plötz, 1884), is reinstated as a species-level taxon.

### Neotype designation for *Hesperia irma* Plötz, 1882

**Figure 6.**
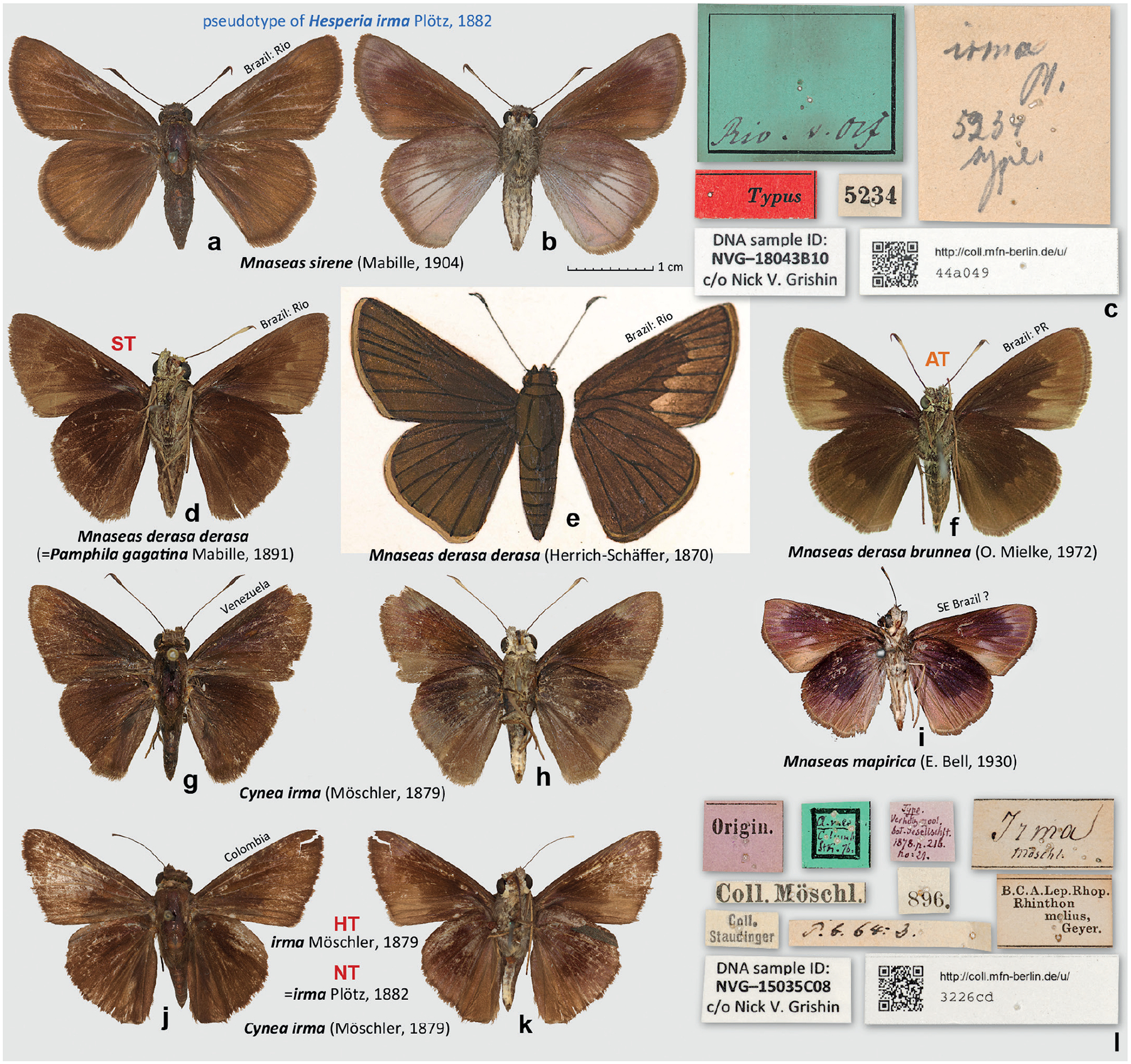
*Hesperia irma* Plötz, 1882. **a–c**. Pseudotype of *Hesperia irma*, specimen number 5234 in MfN, which is *Mnaseas sirene*: **a**. Dorsal view; **b**. Ventral view; **c**. Labels; **d**. A syntype of *Pamphila gagatina* Mabille, 1891, currently a junior subjective synonym of *Mnaseas derasa derasa*, venter, Brazil: Rio de Janeiro [MfN]; **e**. Godman’s copy of the unpublished Plötz’s drawing No. 269 of *Cobalus derasa*; **f**. Allotype of *Mnaseas derasa brunnea* (O. Mielke, 1972), venter; **g**. Dorsal and **h**. Ventral views of *Cynea irma* Venezuela: Aragua, Rancho Grande, 1100 m, 12-Jun-1985, S. S. Nicolay [USNM]; **i**. *Mnaseas mapirica*, no locality label, could be from SE Brazil [BMNH]; **j–l**. the holotype of *Pamphila irma* Möschler, 1879, which is simultaneously the neotype of *Hesperia irma* Plötz, 1882 designated in this work: **j**. Dorsal view; **k**. Ventral view; **l**. Labels. All images are to scale. Photographs **e**. (by N.V.G.) and **i**. (by Bernard Hermier) are Copyright of the Trustees of the Natural History Museum London and are made available under Creative Commons License 4.0 (https://creativecommons.org/licenses/by/4.0/).

In his description of *Hesperia irma* (type locality Colombia), Plötz referred to “Mus. Berol. n. 5234” (Plötz 1882a). The specimen in the MfN collection with No. 5234 (Fig. 6a–c) is curated as “Typus” of *H. irma*, but it shows a number of discrepancies with the original description. The original description was given as a key, relevant sections of which are assembled and translated here: “Antenna – almost always – more than 1/2 as long as the forewing. Wings without hyaline spots. Upperside dark brown or black. Forewing below, past the middle, with a pale, basewards sharply and jaggedly delimited smudge. Hindwing at outer margin and anal margin wide violaceous-grey. 19. *Irma* Pl. Hesp. t. 270. – Mus. Berol. n. 5234. 17 mm. Columbia.” The specimen No. 5234 is significantly (by more than 1/5) larger than described (20.5 mm, not 17 mm), its antenna is shorter than half of the forewing, the ventral forewing pattern is different from that of *Mnaseas derasa derasa* (Herrich-Schäffer, 1870), which Plötz paired with *H. irma* by this character (see below), the outer margin of the ventral hindwing is actually brown, not violaceous-grey, but a violaceous-grey triangle covers nearly half of the wing in the postdiscal area, before the submarginal area (=Saum), and the specimen is from “Rio” according to its label, not from Colombia, collected by v[on]. Olf[ers]. There are a number of other specimens in MfN collected by von Olfers, and they are mostly labelled from “Rio” as well, and none from Colombia. Ignaz Franz Werner Maria von Olfers (1793–1871) must have collected these specimens in Rio de Janeiro when he was there in 1816 as a German diplomat (Wikipedia contributors 2022). This specimen No. 5234 would be identified currently as *Mnaseas sirene* (Mabille, 1904).

Godman did not mention Plötz’s drawing No. 270 in his work (Godman 1907) and a copy of this drawing was not made. However, a copy of *Cobalus derasa* Herrich-Schäffer, 1870 (which Plötz incorrectly synonymised with *Hesperia cassander* Fabricius, 1793) drawing No. 269, a species that Plötz paired with *H. irma* in his key, was available (Fig. 6e). According to Plötz, both *C. derasa* and *H. irma* share the pattern of the ventral forewing, which is “past the middle with a pale, basewards sharply and jaggedly delimited smudge.” This pattern with a well-delimited jagged basal border is apparent from the copy of the drawing No. 269 and is indeed present in many specimens currently identified as *M. derasa* (Fig. 6d, f). However, this pattern is missing in the specimen No. 5234. We also note that the antennae of *C. derasa*, as drawn by Plötz, are longer than half of the forewing, agreeing with the description, not shorter than half, as in the specimen No. 5234 (Fig. 6a, b).

Then, we asked the question: how would an ideal match to the original description of *H. irma* look? Searching many different collections and butterfly images, we came up with a good option (Fig. 6i). Finding this option is a purely theoretical exercise and would not mean that this exact species is conspecific with *H. irma* because there could be a number of candidate species; some may not even be closely related to each other, only sharing similar appearance. However, we think that it may be useful to present various specimens that may fit the original description of *H. irma* rather well. This specimen, identified as *Mnaseas mapirica* (E. Bell, 1930) in the collection of BMNH from an unspecified locality (possibly from SE Brazil or Bolivia), possesses all the characters mentioned in the description of *H. irma*. Its ventral forewing has a smudge sharply delimited basad, and the ventral hindwing is broadly violaceous-grey (=veilgrau) along the outer margin and anal margin. Here, we remind that Plötz used the term “veilgrau” loosely because he also described the mostly pale-brown colour of the *Cecropterus* (*Murgaria*) *jalapus* (Plötz, 1881) ventral hindwing as “veilgrau” (see discussion above). Möschler would probably call this colour violaceous-red (veil-roth) (Möschler 1879), a term that Plötz never used, according to our searches. This *M. mapirica* specimen demonstrates that it is possible to find a fit to the original description of *H. irma* that is nearly ideal and is better than the specimen No. 5234. However, *M. mapirica* is a southern species not recorded in Colombia, the type locality of *H. irma*.

Next, we looked for a species in Colombia that matched the original description of *H. irma*. Such a species, widely distributed over both Americas, is apparently *Pamphila irma* Möschler, 1879 (type locality Colombia), currently in the genus *Cynea* Evans, 1955 (Fig. 6g–l). No other options revealed themselves. Notably, the ventral hindwing submarginal area of many *Cynea irma* specimens can be called “veilgrau”: it is quite similar to the colour in *Polygonus savigny* (Latreille, [1824]), which Plötz described as veilgrau (Plötz 1882a). Even the holotype of *P. irma* Möschler (forewing length 17.5 mm, Fig. 6j–l), also in MfN, is a reasonable match to the description of *H. irma* Plötz, although the specimen is worn and damaged.

For all these reasons, we conclude (1) that the specimen No. 5234 in MfN, which is a female of *Mnaseas sirene* (Mabille, 1904), is not a syntype of *H. irma* Plötz, and (2) that according to its description, supplemented with unpublished drawings of similar-looking species, *H. irma* Plötz is most likely conspecific with *Pamphila irma* Möschler, 1879. Indeed, *Hesperia irma* is one of many taxa described by both Möschler and Plötz, and it frequently is a challenge to figure out whether the specimens used for both descriptions were the same, at least in part. Möschler was sending specimens to Plötz for identification (Möschler 1876), and Plötz might have provided his yet unpublished names to Möschler (or the other way around), drawn these specimens, and used them later in his descriptions. The discrepancies between the specimen No. 5234 and the original description of *H. irma* Plötz, and the agreement of the holotype of *P. irma* Möschler, 1879 with the original description of *H. irma* Plötz suggest that in this instance, at least part of the type material may have been shared by Plötz and Möschler, e.g., it is possible that the holotype of *P. irma* Möschler, 1879 was also a syntype of *H. irma* Plötz. And the specimen that bears the label with the No. 5234, which is from “Rio” and not from the type locality of both *P. irma* Möschler, 1879 and *H. irma* Plötz (“Columbia”), might have been identified as *H. irma* Plötz later, possibly by subsequent workers who could have switched the labels between specimens, especially if the original specimen No. 5234 was damaged.

To correct these inconsistencies, we searched for syntypes of *H. irma* in the collections that are known to house Plötz’s types: MfN, ZSMC and ZIMG. Except for *P. irma* Möschler, 1879 holotype in MfN, which we could not convincingly support as a syntype of *H. irma* Plötz, we failed to find any candidate specimens and believe that the syntypes of *H. irma* are no longer extant. There is an exceptional need to designate a neotype of *H. irma* because a specimen curated as a syntype of this name and bearing a label with the number mentioned in the original description is not a syntype: it does not agree with the original description and the type locality given for this taxon, creating a potential for instability of nomenclature leading to unnecessary nomenclatural changes. To stabilise the nomenclature, we hereby designate the holotype of *Pamphila irma* Möschler, 1879, a male, as the neotype of *Hesperia irma* Plötz, 1882, making the two names objective synonyms and thus keeping the latter as a junior secondary homonym of the former. This neotype is consistent with both the current usage of the name and the original description of this taxon.

This neotype of *H. irma* satisfies all requirements set forth by the ICZN Article 75.3. Requirement 75.3.1: It is designated to clarify the taxonomic identity of this taxon, which may be threatened by a specimen of *Mnaseas sirene* (Mabille, 1904) curated as a syntype and bearing a label with the number referred to in the original description of *H. irma*. Requirement 75.3.2: The characters for the taxon have been given in its original description by Plötz (1882a) and are re-stated above. Requirement 75.3.3: The neotype specimen bears the following 11 labels: || Origin. || A. mer. | Columb. | Sth. 76. || Type | Verhdlg. zool. | bot. Gesellschaft. | 1878. p. 216. | no:21. || Coll. Möschl. || *Irma* | Möschl: || P. b. 64: 3. || 896. || Coll. | Staudinger || B.C.A.Lep.Rhop. | *Rhinthon* | *melius*, | Geyer. || \QR code\ http://coll.mfn-berlin.de/u/ | 3226cd || DNA sample ID: | NVG-15035C08 | c/o Nick V. Grishin ||. Requirement 75.3.4: Our unsuccessful search for the syntypes is described above, leading us to conclude that they are lost. Requirement 75.3.5: as detailed above, the neotype is consistent with the original description of this taxon, other information, such as unpublished drawings, and the current synonymy of this name. Requirement 75.3.6: the neotype is from Colombia, and the type locality given for *H. irma* in the original description is “Columbia.” Requirement 75.3.7: the neotype is in the collection of the Museum für Naturkunde, Berlin, Germany (MfN).

**Figure 7.**
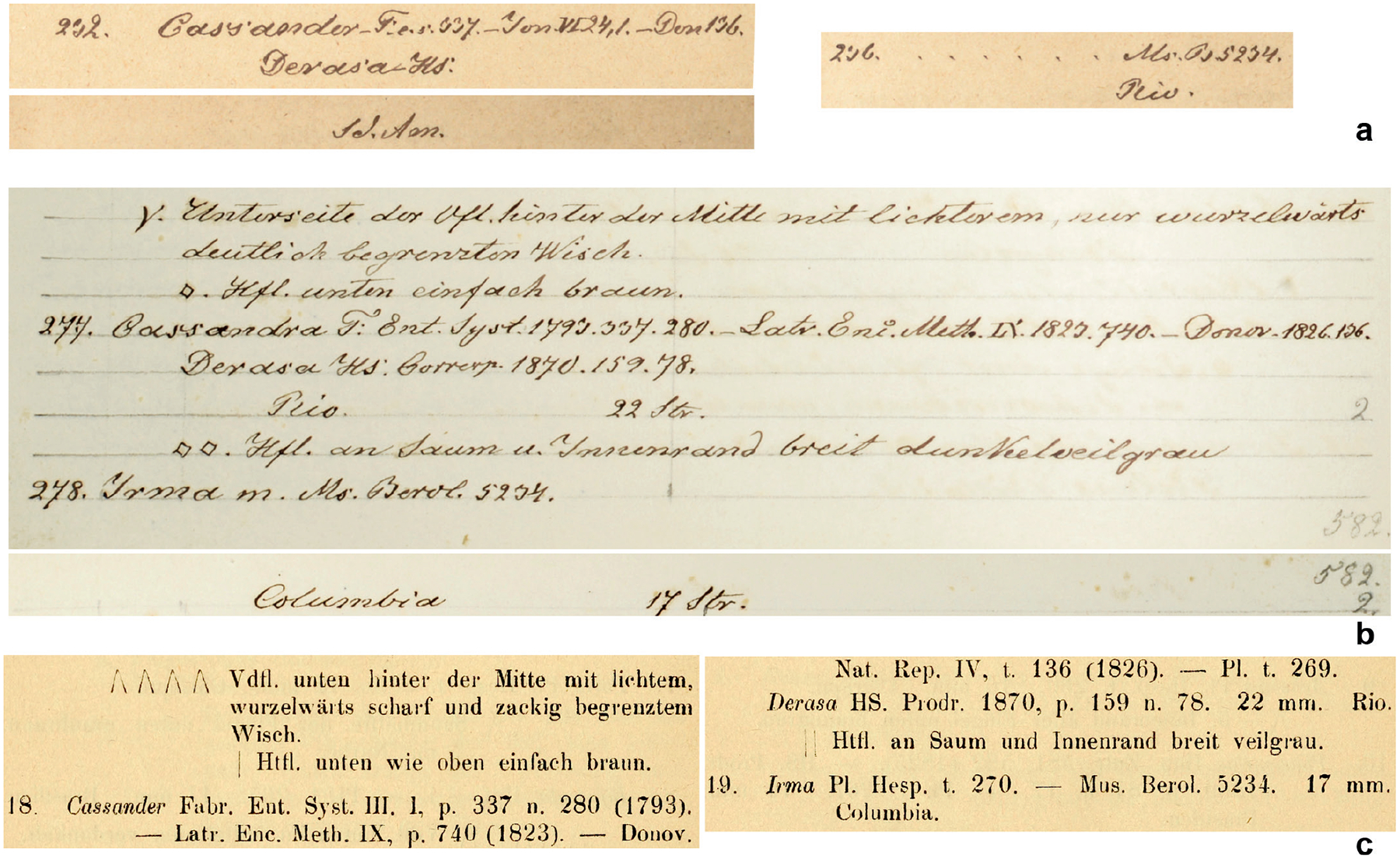
The evolution of Plötz’s writing about the specimen No. 5234 and *Hesperia irma*. **a**. Unpublished list of Hesperiidae dated 1870, in ZSMC; **b**. Unpublished version of the identification key dated 1876, in ZSMC; **c**. Published version, page 316 (Plötz 1882a).

Finally, curious to learn more about the evolution of Plötz’s concept of *H. irma* before the publication of the name, we consulted Plötz’s archive in ZSMC. In his list of Hesperiidae species and specimens known to him, dated 1870, there is an entry “236. …… Ms.B 5234. / Rio.” where the dots occupy the place where Plötz wrote species names (Fig. 7a). E.g., nearby, there is an entry “232. Cassander –F: e.s. 337. –Jon VI 24,1. –Don 136. / Derasa –HS: /Sd.Am.”, a species, which was later placed next to *H. irma* in the published key (Fig. 7a). It seems that in 1870, Plötz knew about the specimen 5234 and that it was from Rio, but he was not able to place a name on it yet. Curiously, a series of specimens under No. 5233 in MfN are *M. derasa derasa*.

Inspecting Plötz’s manuscript containing an early version of his keys, dated 1876, we find *derasa* and *irma* next to each other (numbers 277 and 278) (Fig. 7b), just like in the published version (Fig. 7c). And No. 5234 is mentioned again, but the locality was changed from “Rio” to “Colombia.” The locality for *derasa* became “Rio” instead of South America as in the 1870 list. The key itself matches quite closely the published version, however, with one notable difference. The colour of the ventral hindwing marginal areas is described as “dunkelveilgrau”, not simply “veilgrau”. Dark violet grey is a better description of the colour in the neotype of *H. irma*, which is from Colombia. The manuscript was written in 1876, and the neotype was collected in 1876. We suspect that it was around that time when Plötz included specimens looking like *Pamphila irma* Möschler, 1879 (from Colombia) in his concept of *H. irma*, possibly prioritising them because “Rio” was changed to “Columbia” that remained the type locality in the published version of the key, but “dark” in the colour description was dropped. The number 5234 was retained, but due to all the reasons discussed in this section above, it is not likely that the specimen from Rio was included in Plötz’s final concept of *H. irma*, and it is not likely that the original drawing, and therefore, the description were based on this specimen.

### Questioning the type status of specimens curated as types

One final observation is that all five cases analysed in this study involve specimens from the MfN collections bearing green labels, likely written by Hopffer. These labels are found on the Deppe specimens (larger, nearly square) and Hopffer specimens (smaller, elongated). At least some of these labels were used as header labels corresponding to a series of specimens because two different localities may be found on these labels. Currently, the green label is pinned to one specimen, possibly the header specimen of the series. The reasons for the multiple discrepancies between the appearance of these specimens, their labels, and the original descriptions remain unclear. It is possible that some of them result from either switching labels between specimens or placing these labels on specimens somewhat similar to, but different from, those used by Plötz for his descriptions and drawings. Therefore, we suggest being more careful in determining the actual type status of the specimens in MfN, particularly those bearing green labels. We encourage researchers to analyse each case individually and thoroughly, thus finding a reasonable compromise between the stability of nomenclature and the perceived intent of the original author expressed in the description and interpreted in subsequent studies.

## References

[R1] Cong Q, Shen J, Zhang J, Li W, Kinch LN, Calhoun JV, Warren AD, Grishin NV (2021) Genomics reveals the origins of historical specimens. Molecular Biology and Evolution 38(5): 2166–2176. 10.1093/molbev/msab013 33502509 PMC8097301

[R2] Dolibaina DR, Mielke OH, Casagrande MM (2014) Taxonomic revision of *Cumbre* Evans, 1955 (Hesperiidae: Hesperiinae: Moncini), with the description of two new species. Zootaxa 3841(1): 47–66. 10.11646/zootaxa.3841.1.2 25082027

[R3] Draudt MWK (1921–1924) B. Grypocera, breitköpfige Tagfalter. In: Seitz A (Ed.) Die Gross-Schmetterlinge der Erde 5. Alfred Kernen, Stuttgart, 833–1011. [1046–1139, pls 1113B, 1160–1193.]

[R4] Evans WH (1937) A Catalogue of the African Hesperiidae Indicating the Classification and Nomenclature Adopted in the British Museum. The Trustees of the British Museum (Natural History), London, 212 pp. [230 pls.]

[R5] Evans WH (1943) A revision of the genus *Aeromachus* de N. (Lepidoptera: Hesperiidae). Proceedings of the Royal Entomological Society of London (B) 12(7–8): 97–101. 10.1111/j.1365-3113.1943.tb00751.x

[R6] Evans WH (1949) A Catalogue of the Hesperiidae from Europe, Asia, and Australia in the British Museum (Natural History). The Trustees of the British Museum (Natural History), London, 502 pp. [553 pls.] 10.5962/bhl.title.105941

[R7] Evans WH (1951) A Catalogue of the American Hesperiidae Indicating the Classification and Nomenclature Adopted in the British Museum (Natural History). Part I. Introduction and Group A Pyrrhopyginae The Trustees of the British Museum (Natural History), London, 92 pp. [pls. 91–99.]

[R8] Evans WH (1952) A Catalogue of the American Hesperiidae Indicating the Classification and Nomenclature Adopted in the British Museum (Natural History). Part II. Pyrginae. Section I The Trustees of the British Museum (Natural History), London, 178 pp. [pls. 110–125.]

[R9] Evans WH (1953) A Catalogue of the American Hesperiidae indicating the Classification and Nomenclature Adopted in the British Museum (Natural History). Part III. Pyrginae. Section 2 The Trustees of the British Museum (Natural History), London, 246 pp. [pls. 226–253.]

[R10] Evans WH (1955) A Catalogue of the American Hesperiidae Indicating the Classification and Nomenclature Adopted in the British Museum (Natural History). Part IV. Hesperiinae and Megathyminae The Trustees of the British Museum (Natural History), London, 499 pp. [pls. 454–488.]

[R11] Godman FD (1907) Notes on the American species of Hesperiidae described by Plötz. Annals & Magazine of Natural History 20(7): 132–155. 10.1080/00222930709487316

[R12] ICZN [International Commission on Zoological Nomenclature] (1999) International code of zoological nomenclature (4^th^ edn.). International Trust for Zoological Nomenclature, London, 306 pp. 10.3897/zookeys.931.51583 PMC7205856 32405237

[R13] Li W, Cong Q, Shen J, Zhang J, Hallwachs W, Janzen DH, Grishin NV (2019) Genomes of skipper butterflies reveal extensive convergence of wing patterns. Proceedings of the National Academy of Sciences of the United States of America 116(13): 6232–6237. 10.1073/pnas.1821304116 30877254 PMC6442542

[R14] Mielke OHH (2005) Catalogue of the American Hesperioidea: Hesperiidae (Lepidoptera). Sociedade Brasileira de Zoologia, Curitiba, Paraná, Brazil, 1536 pp.

[R15] Mielke OHH, Casagrande MM (2002) Notas taxonômicas em Hesperiidae neotropicais, com descrições de novos taxa (Lepidoptera). Revista Brasileira de Zoologia 19(suppl 1): 27–76. 10.1590/S0101-81752002000500002

[R16] Möschler HB (1876) Beiträge zur Schmetterlings-fauna von Surinam. Verhandlungen der kaiserlich-königlichen zoologisch-botanischen Gesellschaft in Wien 26: 293–352.

[R17] Möschler HB (1879) Neue exotische Hesperiidae. Verhandlungen der kaiserlich-königlichen zoologisch-botanischen Gesellschaft in Wien 28: 203–230.

[R18] Plötz C (1879a) Die Hesperiinen-Gattung *Erycides* Hübn. und ihre Arten. Stettiner entomologische Zeitung 40: 406–411. [(410/412): 474.]

[R19] Plötz C (1879b) Die Hesperiinen-Gattung Pyrrhopyga und ihre Arten. Stettiner entomologische Zeitung 40: 520–538.

[R20] Plötz C (1879c) Hesperiina Herr. Sch. Stettiner entomologische Zeitung 40: 175–180.

[R21] Plötz C (1881a) Die Hesperiinen-Gattung *Eudamus* und ihre Arten. Stettiner entomologische Zeitung 42: 500–504. [543(501/503): 587–101.]

[R22] Plötz C (1881b) Die Hesperiinen-Gattung *Goniurus* Hüb. und ihre Arten. Bulletin de la Société impériale des Naturalistes de Moscou 55: 1–22.

[R23] Plötz C (1882a) Die Hesperiinen-Gattung *Hesperia* Aut. und ihre Arten. Stettiner entomologische Zeitung 43: 314–344. 10.1002/mmnd.47918820406

[R24] Plötz C (1882b) Die Hesperiinen-Gattung *Hesperia* Aut. und ihre Arten. Stettiner entomologische Zeitung 43: 314–344. [(310/312): 436–456; 344(311/313): 326–364.]

[R25] Plötz C (1882c) Einige Hesperiinen-Gattungen und deren Arten. Berliner Entomologische Zeitschrift 26: 253–266. 10.1002/mmnd.47918820406

[R26] Plötz C (1882d) Einige Hesperiinen-Gattungen und deren Arten. Berliner Entomologische Zeitschrift 26: 71–82. 10.1002/mmnd.47918820306

[R27] Plötz C (1883a) Die Hesperiinen-Gattung *Entheus* Hüb. und ihre Arten. Stettiner entomologische Zeitung 44: 456–458.

[R28] Plötz C (1883b) Die Hesperiinen-Gattung *Hesperia* Aut. und ihre Arten. Stettiner entomologische Zeitung 44: 195–233.

[R29] Plötz C (1883c) Die Hesperiinen-Gattung *Ismene* Sw. und ihre Arten. Stettiner entomologische Zeitung 45: 51–66.

[R30] Plötz C (1883d) Die Hesperiinen-Gattung *Phareas* Westw. und ihre Arten. Stettiner entomologische Zeitung 44: 451–456.

[R31] Plötz C (1884a) Analytische Tabellen der Hesperiinen-Gattungen *Pyrgus* und *Carcharodus*. Mittheilungen aus dem naturwissenschaftlichen Vereine von Neu-Vorpommern und Rügen in Greifswald 15: 1–24.

[R32] Plötz C (1884b) Die Gattung *Cyclopides* Hüb. und ihre Arten. Stettiner entomologische Zeitung 45: 389–397.

[R33] Plötz C (1884c) Die Hesperiinen-Gattung Apaustus Hüb. und ihre Arten. Stettiner entomologische Zeitung 45: 151–166.

[R34] Plötz C (1884d) Die Hesperiinen-Gattung Butleria Kirby und ihre Arten. Stettiner entomologische Zeitung 45: 290–295.

[R35] Plötz C (1884e) Die Hesperiinen-Gattung Carterocephalus Led. und ihre Arten. Stettiner entomologische Zeitung 45: 386–388.

[R36] Plötz C (1884f) Die Hesperiinen-Gattung Plastingia Butl. und ihre Arten. Stettiner entomologische Zeitung 45: 145–150.

[R37] Plötz C (1884g) Die Hesperiinen-Gattung Telesto Bsd. und ihre Arten. Stettiner entomologische Zeitung 45: 376–384.

[R38] Plötz C (1884h) Die Hesperiinen-Gattung Thymelicus Hüb. und ihre Arten. Stettiner entomologische Zeitung 45: 284–290.

[R39] Plötz C (1884i) Die Hesperiinen-Gruppe der Achlyoden. Jahrbücher des nassauischen Vereins für Naturkunde 37: 1–55.

[R40] Plötz C (1885) Die Hesperiinen-Gattung *Leucochitonea* Wlgr.? und ihre Arten. Stettiner entomologische Zeitung 46: 36–40.

[R41] Plötz C (1886) Nachtrag und Berichtigungen zu den Hesperiinen. Stettiner entomologische Zeitung 47: 83–117.

[R42] Wikipedia contributors (2022) Ignaz von Olfers. Wikipedia, The Free Encyclopedia, 31 August 2022, 01:54 UTC. https://en.wikipedia.org/w/index.php?title=Ignaz_von_Olfers&oldid=1107636950 [accessed 19 September 2022]

[R43] Zhang J, Cong Q, Shen J, Opler PA, Grishin NV (2021) Genomics-guided refinement of butterfly taxonomy. The Taxonomic Report of the International Lepidoptera Survey 9: 1–54. 10.5281/zenodo.5630311 PMC8794009 35098146

